# Telomere length and *TERT* polymorphisms as biomarkers in asbestos-related diseases

**DOI:** 10.2478/raon-2024-0009

**Published:** 2024-02-21

**Authors:** Ana Mervic, Katja Goricar, Tanja Blagus, Alenka Franko, Katarina Trebusak-Podkrajsek, Metoda Dodic Fikfak, Vita Dolzan, Viljem Kovac

**Affiliations:** Faculty of Medicine, University of Ljubljana, Ljubljana, Slovenia; Institute of Biochemistry and Molecular Genetics, Faculty of Medicine, University of Ljubljana, Ljubljana, Slovenia; Clinical Institute of Occupational Medicine, University Medical Centre Ljubljana, Ljubljana, Slovenia; Clinical Institute for Special Laboratory Diagnostics, University Children’s Hospital, University Medical Centre Ljubljana, Ljubljana, Slovenia; Institute of Oncology Ljubljana, Ljubljana, Slovenia

**Keywords:** malignant mesothelioma, asbestos, telomere length, *hTERT* polymorphisms

## Abstract

**Background:**

Asbestos exposure has been proposed as a risk factor for shorter telomere length. The aim of our study was to investigate whether telomere length in leukocytes and *hTERT* genetic polymorphisms may serve as potential biomarkers for the risk of developing asbestos-related diseases and as biomarkers of progression and chemotherapy response rate in malignant mesothelioma (MM).

**Subjects and methods:**

We conducted two retrospective studies. In the first study, a case-control study, telomere length and *hTERT* polymorphisms were determined in patients with MM, subjects with pleural plaques and controls without the asbestos related disease, who were occupationally exposed to asbestos. In the second study, a longitudinal observational study, telomere length was also determined in samples from MM patients before and after chemotherapy. Telomere length was determined by monochromatic multiplex quantitative polymerase chain reaction (PCR), while competitive allele-specific PCR was used to genotype *hTERT* rs10069690, rs2736100 and rs2736098. Logistic regression and survival analysis were used in statistical analysis.

**Results:**

Patients with MM had shorter telomere length than subjects with pleural plaques (p < 0.001). After adjustment for age, rs2736098 CT, and rs10069690 TT and CT+TT genotypes were significantly associated with a higher risk of MM (p_adj_ = 0.023; p_adj_ = 0.026 and p_adj_ = 0.017), while rs2736100 AA and CA+AA genotypes conferred to a lower risk for MM compared to all other subjects (p_adj_ = 0.017, and p_adj_ = 0.026). Telomere length was not associated with a response to chemotherapy (p > 0.05) or time to disease progression (p > 0.05). Carriers of one or two polymorphic rs10069690 T alleles had a good response to chemotherapy (p = 0.039, and p = 0.048), these associations remained statistically significant after adjustment for age (p_adj_ = 0.019; p_adj_ = 0.017). Carriers of two polymorphic rs2736100 A alleles had a longer time to disease progression (p = 0.038).

**Conclusions:**

Shorter telomere length and *hTERT* polymorphisms may serve as a biomarker for the risk of developing MM. Additionally, rs10069690 and rs2736100 polymorphisms, but not telomere length, were associated with a chemotherapy response or MM progression.

## Introduction

Asbestos consists of mineral fibres with a high malignant potential, listed among carcinogens by the World Health Organisation in 1987.^1^ The malignant potential of asbestos fibres lies within their capability to deeply infiltrate the respiratory system and persist there for extended durations.^2^ There is no known safe level of asbestos exposure.^3^

Inhaled asbestos fibres induce oxidative stress due to the presence of iron as well as frustrated phagocytosis by macrophages, what in turn stimulates Fenton and Haber-Weiss reactions, ultimately resulting in the generation of reactive oxygen species (ROS). Asbestos further elicits the upregulation of the heavy chain of ferritin, consequently leading to an augmented iron burden and increased production of ROS.^5^ Oxidative stress is closely associated with chronic inflamation. The asbestos deposits contribute to the production of iron-rich asbestos bodies, that are accountable for sustaining chronic inflamation.^4^ The pro-inflamatory microenvironment fosters cell survival by inhibiting apoptosis, promoting mesothelial cell proliferation despite DNA damage, activating fibroblasts and inducing immunosupression.^2,5^ Furthermore, asbestos fibres also have a detrimental impact on chromosomes. All of these mechanisms eventually contribute to the induction of carcinogenesis.^6,7^

Asbestos exposure causes several diseases. In the lung, asbestosis and lung cancer carcinoma affect the lung parenchyma, while pleural plaques (PP) and malignant mesothelioma (MM) affect the pleura, but other serous membranes such as peritoneum may also be affected.^8^

Malignant mesothelioma arises from the malignant transformation of mesothelial cells and is a rare, yet highly aggressive cancer with a poor prognosis. In over 80% of cases, the development of MM is associated with asbestos exposure.^9^ The majority of patients who develop MM have been exposed to asbestos through occupational exposure; however, para-occupational, domestic and environmental exposure have also been associated with MM development.^8^ Due to its long latency period, which can extend up to 40 years or even longer, the MM epidemic continues to rise in Central Europe.^8,10,11^ Malignant mesothelioma occurs more often in males, with a median age of 70 years.^3^ In 2018, the incidence of MM in Slovenia was 3.0/100 000 in males and 1.5/100 000 in females, resulting in a total of 45 new cases that year. At the time of diagnosis, 31.1% of patients had MM in an early localized stage, 55.6% had cancer spread to lymph nodes and in 11.1% it already presented with metastasis.^12^ Malignant mesothelioma can be histologically classified into subgroups: epithelioid, biphasic and sarcomatoid subtypes. The epithelioid subtype is the most common and also exhibits the most favourable prognosis, while patients with sarcomatoid MM tend to have the worst prognosis.^9^

The diagnosis of MM is accomplished through clinical examination, thorax CT or MRI, and PET-CT. The prevailing clinical presentation often involves progressive dyspnoea, accompanied by non-pleuritic chest pain. Additional symptoms include cough, fever, asthenia, hypoxia, weight loss, and night sweats. Typically, the disease is detected 3–6 months after the initial clinical presentation.^2^ The therapeutic strategy is tailored based on the tumour resectability and the patient’s performance status. In resectable disease, the treatment involves the combination of surgery, chemotherapy and radiotherapy, while patients with unresectable disease receive systemic treatment. Chemotherapy with pemetrexed/cisplatin doublet has not been changed as a standard treatment since 2004 and immunotherapy with ipilimumab and nivolumab was approved for the first line treatment in late 2020.^13^ Although different chemotherapy (gemcitabine/cisplatin, pemetrexed single, vinorelbine weekly) and immunotherapy (nivolumab/ipilimumab) regimens are used in relapsed MM, there is still no standard of care.^2,13,14^

The increasing incidence of MM and the poor prognosis call for the identification of novel noninvasive biomarkers that will enable an earlier diagnosis, will have a prognostic value and/or will predict the response to treatment. Despite the growing numbers of potential biomarkers, there is no reliable diagnostic or prognostic biomarker available yet. Telomerase reactivation may play a crucial role in cancer development and progression, and telomeres could serve both as a potential biomarker and as a therapeutic target in cancer. In somatic cells telomeres shorten with each cell division, ultimately leading to senescence or apoptosis.^15,16^ Cancer cells gain the ability to sustain their telomere length by reactivating telomerase, a process typically suppressed under physiological circumstances.^15,16,17,18^

The regulation of the expression of the human telomerase reverse transcriptase (hTERT) subunit of telomerase occurs predominantly at the transcriptional level.^19,20,21^ Numerous single nucleotide polymorphisms (SNP) in the *hTERT* gene may also have an impact on telomerase expression levels and activity, and may thus play a role in the risk of carcenogenesis, as well as the prognosis and survival of cancer patients.^18,19,22^

Telomere length is influenced by cellular senescence, chronic inflammation and oxidative stress. Telomere shortening itself serves as one of the main markers for senescence, as telomeres typically shorten by 50–200 base pairs (bp) with each cell cycle.^23^ Chronic inflammation is associated with elevated *hTERT* expression, which, in turn, maintains telomere length.^7^ On the other hand, the celullar turnover stimulated by chronic inflamation leads to an increased number of cell divisions, resulting in telomere shortening.^19^ Furthermore, oxidative stress causes DNA damage, which subsequently stops telomere elongation.^7,18^

Cancer cells exhibit shorter, yet stable, telomeres compared to non-neoplastic cells.^18,20,21^ Malignant mesothelioma is considered to be a telomerase dependant cancer.^22^ The impact of asbestos exposure on telomere length was also established in pleural effusion cells, showing that non-neoplastic cells had longer telomeres than neoplastic MM cells and that telomere shortening and genomic instability play significant roles in MM pathogenesis, and may also serve as biomarkers for disease development, treatment response, and prognosis.^23^

To the best of our knowledge, the association between telomere length and *hTERT* polymorphisms and asbestos-related diseases has not been evaluated yet. Thus, the aim of the present study was to analyse the role of telomere length in leukocytes and *hTERT* polymorphisms as a biomarker for asbestos-related diseases, in particular MM, its response to treatment and prognosis.

## Subjects and methods

### Subjects

We conducted two retrospective studies. In the first study, a case-control study, telomere length and *hTERT* polymorphisms were determined in 340 patients with MM, 380 subjects with pleural plaques and 94 control subjects without any diseases related to asbestos exposure. The cases with PP and controls had a history of occupational asbestos exposure while working at the Salonit Anhovo factory, Slovenia and were presented before the State Board for the Recognition of Occupational Asbestos Diseases between January 1999 and December 2003. The patients with MM were treated at the Institute of Oncology Ljubljana from 2008 and 2018. Among MM patients, 94 had blood samples available from at least two different time points during chemotherapy treatment (211 samples available in total).

The diagnosis of MM, PP, or “no asbestos related disease” was confirmed by the experts of the State Board for the Recognition of Occupational Asbestos Diseases.^24,25,26^ In all subjects of the study high-resolution computed tomography (HRCT) was performed. Pleural MM was histologically confirmed based on samples obtained through thoracoscopy or video-assisted thoracic surgery, while samples for confirming peritoneal MM were collected via laparascopy. The histopathologic samples were classified as epithelioid, sarcomatoid, biphasic or undifferentiated types of MM.^2,27,28^ The TNM classification was used for staging pleural MM.^29^ Additionally, clinical data on MM patients, such as a performance status based on The Eastern Cooperative Oncology Group (ECOG), weight loss and C-reactive protein (CRP) levels were also collected.

Data on the chemotherapy protocol (gemcitabine with cisplatin, pemetrexed with cisplatin, other, or no chemotherapy) and chemotherapy response (classified as complete response [CR], partial response [PR], stable disease [SD], progressive disease [PD]) were collected from patients’ medical records at The Institute of Oncology Ljubljana and the Cancer Registry of the Republic of Slovenia.

Data on asbestos exposure were available from our previous studies.^30^ A standardized questionnaire-based interview was conducted with cases having PP and control group to gather data on their smoking status, whereas data for patients with MM was extracted from medical records at The Institute of Oncology Ljubljana.^31^

All participants were fully informed about the purpose of the study and willingly provided their informed written consent to participate. The study was part of the comprehensive studies approved by the Slovenian Ethics Committee for Research in Medicine (KME 41/02/09, 36/02/04 and 31/07/04). The study adhered to the principles outlined in the Declaration of Helsinki.

### Molecular genetic analysis

Peripheral venous blood samples from MM patients were collected in Tempus tubes and frozen at −80°C until the analysis. DNA extraction was performed using the MagMax 96DNA Multi Sample Kit and the MagMax protocol for stabilized Blood Tub RNA isolation (all, Applied Biosystems [ABI]). For the purpose of our study, only DNA was extracted, while the remaining sample was stored for later RNA extraction. Genomic DNA of cases with PP and control group had been isolated during our previous studies from peripheral venous blood collected in ethylenediaminetetraacetic acid (EDTA) containing tubes and DNA was extracted with the QIAmp DNA Mini Kit (QIAGEN). The concentration of DNA samples was measured using the Perkin Elmer Lambda BIO+ UV/VIS spectrophotometer. Telomere length was assessed using monochrome multiplex quantitative polymerase chain reaction (MMQ-PCR), relatively as the ratio between the telomere product (Tel) and albumin gene product (Alb) as previously described.^32^ Genotyping of *hTERT* rs10069690, rs2736100 and rs2736098 polymorphisms was performed using the competitive allele-specific polymerase chain reaction (KASP) SNP Genotyping Assay (LGC Group).

### Statistics

Descriptive statistics were used to depict the variables. Continuous variables were described using the median and interquartile range, while categorical variables were presented as frequencies. To compare the distribution of continuous variables, the non-parametric Kruskal-Wallis test was performed; for categorical variables, Fisher’s exact test was used.

The association between telomere length and categorical variables was analyzed using the Mann-Whitney test and the Kruskal-Wallis test. The Wilcoxon test for related samples was used to evaluate the longitudinal change in telomere length. Additionally, the correlation between continuous variables and longitudinal telomere length change was assessed using Spearman’s Rho correlation coefficient.

Minor allele frequency (MAF) was analysed for each investigated polymorphism. The deviation from the Hardy-Weinberg equilibrium (HWE) was tested using a chi-square test. A p-value less than 0.05 indicated that the distribution did not adhere to HWE. Both additive and dominant genetic models were used in statistical analyses. Univariate logistic regression was used to assess the association between telomere length and polymorphisms with asbestos-related diseases and the response to chemotherapy.

Cox regression was utilized to evaluate the association of telomere length and genotypes with progression-free survival (PFS) and overall survival (OS). Kaplan-Meier method was used to illustrate the PFS function over time.

All statistical analyses were conducted using the IBM SPSS Statistics, version 27.0 (IBM Corporation, Armonk, NY, USA). The threshold for statistical significance in all tests performed was set at 0.05.

## Results

### Subjects

In total, 302 patients with MM, 386 cases with PP and 86 controls were included in our study. The characteristics of patients with MM, cases with PP and the control group are shown in Table 1. There were statistically significant differences between the groups in respect to age (p < 0.001) and asbestos exposure (p < 0.001). Patients with MM (66.0 (59.0–73.0) years) were significantly older than cases with PP (55.0 (48.8–62.7) years) and control subjects (53.3 (48.1–59.5) years). Asbestos exposure was available for the control group, 379 cases with PP and 42 patients with MM. Among subjects with known asbestos exposure, 52.7% of patients with MM had medium or high exposure, compared to 28.5% of cases with PP and 24.4% of the control group. However, the three study groups did not differ significantly with regards to gender (p = 0.467) and smoking status (p = 0.254) (Table 1).

**TABLE 1.** Characteristics of subjects included in the study

**Characteristics**	**Category/unit**	**Total participants (N = 774)**	**Control group (N = 86)**	**Cases with PP (N = 386)**	**Patients with MM (N = 302)**	**P**
Gender	Male, N (%)	555 (71.7)	63 (73.3)	269 (69.7)	223 (73.8)	0.467^a^
	Female, N (%)	219 (28.3)	23 (26.7)	117 (30.3)	79 (26.2)	
Age	Years, median (25%–75%)	59.1 (51.1–67.5)	53.3 (48.1–59.5)	55.0 (48.8–62.7)	66.0 (59.0–73.0)	**< 0.001^b^**
Smoking	No, N (%)	398 (52.0) [8]	47 (54.7)	189 (49.0)	162 (55.1)	0.254^a^
	Yes, N (%)	368 (48.0)	39 (45.3)	197 (51.0)	132 (44.9)	

Number of missing data is presented in [] brackets. Statistically significant values are printed in bold.

a Calculated using Fisher exact test;

b Calculated using Kruskal-Wallis test.

MM = malignant mesothelioma; N = number of samples; PP = pleural plaques

The clinical characteristics of patients with MM are summarized in Table 2. The majority had pleural MM (267; 88.7%) of the epithelioid type (227; 75.2%) and had stage four (93; 34.8%) or stage three (89; 33.3%) of the disease. According to the ECOG performance status (PS), most patients with MM had PS 1 (154; 51.2%) or PS 2 (111; 36.9%).

**TABLE 2.** Clinical characteristics of patients with malignant mesothelioma (MM) (N = 302)

**Characteristics**	**Category**	**N (%)**
Location [1]	Pleura	267 (88,7)
	Peritoneum	34 (11,3)
Histology type	Epithelioid	227 (75,2)
	Biphasic	27 (8,9)
	Sarcomatoid	27 (8,9)
	Undifferentiated	21 (6,9)
Stage (pleural MM)	1	20 (7,5)
	2	65 (24,3)
	3	89 (33,3)
	4	93 (34,8)
ECOG performance status [1]	0	18 (6,0)
	1	154 (51,2)
	2	111 (36,9)
	3	18 (6,0)
Asbestos exposure [8]	No	79 (26,9)
	Yes	215 (73,1)
Pain [29]	No	114 (41,8)
	Yes	159 (58,2)
Weight loss [34]	No	97 (36,2)
	Yes	171 (63,8)
CRP [mg/mL] [48]	Median (25%–75%)	22 (7–63,5)
Chemotherapy [21]	No chemotherapy	17 (6.1)
	Gemcitabine with cisplatin	161 (57.3)
	Pemetrexed with cisplatin	92 (31.6)
	Other	11 (3.9)
Chemotherapy response [46]	CR	10 (3.9)
	PR	73 (28.5)
	SD	128 (50.0)
	PD	45 (17.6)
Response rate	Poor response (SD+PD)	173 (67.6)
	Good response (PR+CR)	83 (32.4)

Number of missing data is presented in [] brackets.

CRP = C-reactive protein; ECOG = Eastern Cooperative Oncology Group; CR = complete response; N = number of samples; SD = stable disease; PD = progressive disease; PR = partial response

### Telomere length

**FIGURE 1.**
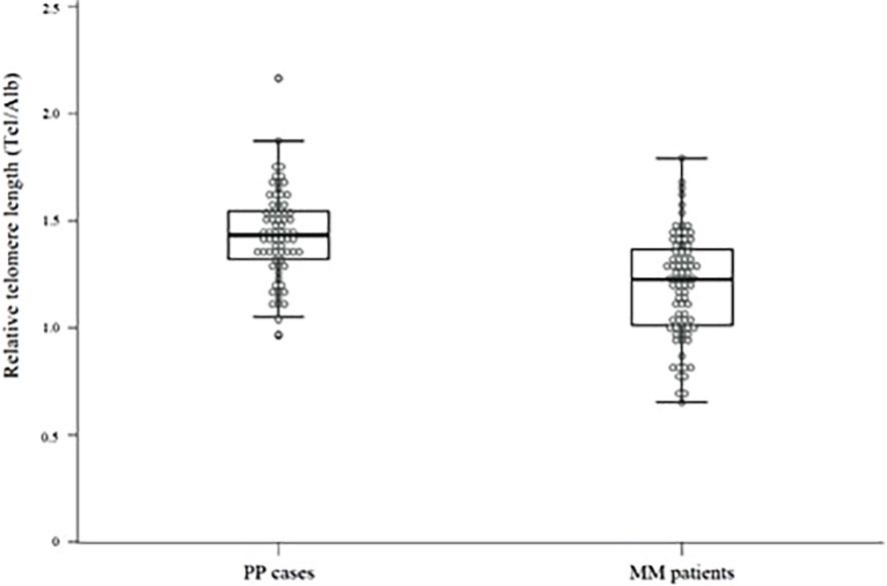
Relative telomere length in cases with pleural plaques (PP) and patients with malignant mesothelioma (MM). Alb = albumin gene product Tel = telomere product

There was a statistically significant difference in telomere length between patients with MM and cases with PP (p < 0.001) (Figure 1). Patients with MM had shorter median telomere length of 1.23 (1.01–1.37) compared to 1.43 (1.32–1.56) in cases with PP. The difference in telomere length remained statistically significant (p < 0.001) after adjustment for age.

The analysis of the association between telomere length and age revealed a statistically significant influence of age on telomere length, indicating that older patients with MM had longer telomeres (Spearman’s rho = 0.370; p < 0.001).

### The dynamics of telomere length during chemotherapy

**FIGURE 2.**
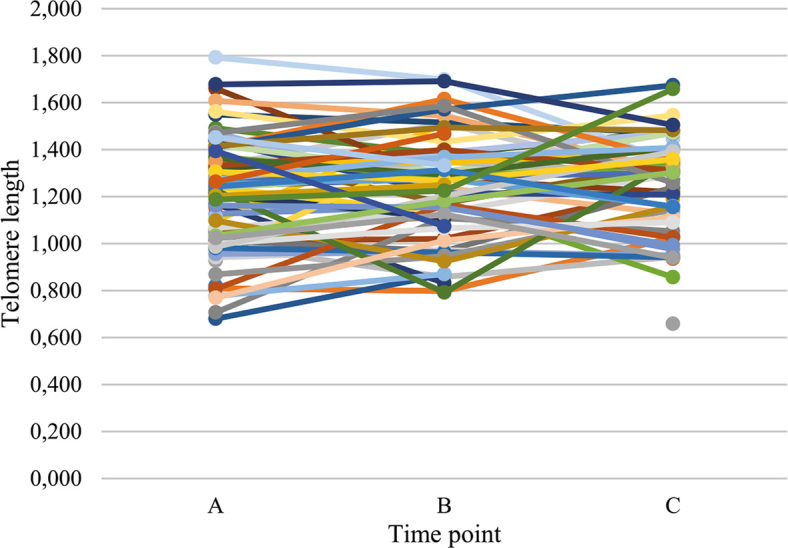
Relative telomere length at different time points A, B and C. A = telomere length before first chemotherapy cycle: B = telomere length at third chemotherapy cycle; C = telomere length after completed chemotherapy or at disease progression

Among the patients with MM, no specific trend was observed in telomere length changes at different time points during chemotherapy. Approximately the same number of cases exhibited telomere elongation or shortening (Table 3, Figure 2).

**TABLE 3.** Telomere length in patients with malignant mesothelioma (MM) at different time points during chemotherapy

**Time point**	**N**	**Median (25%–75%)**	**P**	**N shortens/N prolongs**
A	79	1.23 (1.01–1.37)		
B	66	1.23 (1.10–1.38)		
C	66	1.27 (1.08–1.36)		
Comparison B *vs*. A	66		0.480	28 shortens
				38 prolongs
Comparison C *vs*. A	66		0.423	32 shortens
				34 prolongs
Comparison C *vs*. B	53		0.733	26 shortens
				27 prolongs

A = telomere length before first chemotherapy cycle; B = telomere length at third chemotherapy cycle; C = telomere length after completed chemotherapy or at disease progression; N = number of samples; P = p value

### *hTERT* polymorphisms and the risk for asbestos-related diseases

We investigated three *hTERT* polymorphisms: rs2736098, rs2736100 and rs10069690. The distribution of all genotypes followed the Hardy-Weinberg equilibrium (HWE) (pHWE > 0.05). Genotype frequencies in different study groups are presented in Table 4.

**TABLE 4.** Comparison of genotype frequencies in control group, cases with pleural plaques (PP) and patients with malignant mesothelioma (MM)

		**Control group (N = 86)**	**Cases with PP (N = 386)**	**Patients with MM (N = 302)**	**P**

**SNP**	**Genotype**	**N (%)**	**N (%)**	**N (%)**	
**rs2736098**	CC	45 (56.3)	215 (56.3)	139 (46.8)	**Padd = 0.018**
	CT	28 (35.0)	133 (34.8)	140 (47.1)	
	TT	7 (8.8)	34 (8.9)	18 (6.1)	
	CT+TT	35 (43.8)	167 (43.7)	158 (53.2)	**Pdom = 0.039**
**rs2736100**	CC	17 (20.0)	103 (26.8)	93 (30.8)	Padd = 0.362
	CA	48 (56.5)	194 (50.4)	147 (48.7)	
	AA	20 (23.5)	88 (22.9)	62 (20.5)	
	CA+AA	68 (80.0)	282 (73.2)	209 (69.2)	Pdom = 0.127
**rs10069690**	CC	48 (63.2)	233 (61.0)	160 (54.6)	Padd = 0.107
	CT	26 (34.2)	131 (34.3)	107 (36.5)	
	TT	2 (2.6)	18 (4.7)	26 (8.9)	
	CT+TT	28 (36.8)	149 (39.0)	133 (45.4)	Pdom = 0.178

A = adenine; C = cytosine; N = number of samples; Padd = p value of additive genetic model; Pdom = p value of dominant genetic model; SNP = single nucleotide polymorphism; T = thymine. Statistically significant values are printed in bold.

For further analysis, the most common CC genotype was used as the reference.

For rs2736098, the genotype distribution differed significantly between groups association with MM (P value of additive genetic model [Padd] = 0.018; P value of dominant genetic model [Pdom] = 0.039). Carries of polymorphic rs2736098 T alleles were more common among patients with MM compared to cases with PP and control subjects. There were no significant differences in the distribution of other investigated polymorphisms among the groups (Table 4).

When MM patients were compared to all other subjects combined, polymorphic rs2736098 T allele was statistically significantly associated with an increased risk of developing MM (CT genotype: odds ratio [OR] = 1.63; 95% confidence interval [CI] = 1.20–2.21; p = 0.002; CT+TT genotype: OR = 1.46; CI = 1.09–1.96; p = 0.011) (Table 5). After adjustment for age, only the association of CT genotype remained significant (OR_adj_ = 1.49; CI_adj_ = 1.06–2.10; p_adj_ = 0.023). The presence of at least one polymorphic rs2736100 A allele was associated with a lower risk for developing MM after age adjustment (AA genotype: OR_adj_ = 0.56; CI_adj_ = 0.35–0.90; p_adj_ = 0.017; CA+AA genotype: OR_adj_ = 0.66; CI_adj_ = 0.46–0.95; p_adj_ = 0.026). Carriers of two polymorphic rs10069690 T alleles had a higher risk of MM development (TT genotype: OR = 2.28; CI = 1.24–4.22; p = 0.008). After adjustment for age, the risk for MM was significantly higher in carriers of at least one polymorphic allele (CT+TT genotype: OR_adj_ = 1.52; CI_adj_ = 1.08–2.12; p_adj_ = 0.017) as well as in carriers of two polymorphic alleles (TT genotype: OR_adj_ = 2.2; CI_adj_ = 1.10–4.48; p_adj_ = 0.026) (Table 5).

**TABLE 5.** Association between selected polymorphisms and the risk of developing malignant mesothelioma (MM): comparison of patients with MM and other participants (control group and cases with pleural plaques [PP])

		**Patients with MM**	**Others**				

**SNP**	**Genotype**	**N (%)**	**N (%)**	**OR (95% CI)**	**P**	**OR (95% CI)_adj_**	**P_adj_**
**rs2736098**	CC	139 (46.8)	260 (56.3)	Reference		Reference	
	CT	140 (47.1)	161 (34.8)	1.63 (1.20–2.21)	**0.002**	1.49 (1.06–2.10)	**0.023**
	TT	18 (6.1)	41 (8.9)	0.82 (0.46–1.48)	0.514	0.78 (0.40–1.54)	0.470
	CT+TT	158 (53.2)	202 (43.7)	1.46 (1.09–1.96)	**0.011**	1.36 (0.98–1.88)	0.070
**rs2736100**	CC	93 (30.8)	120 (56.3)	Reference		Reference	
	CA	147 (48.7)	242 (51.5)	0.78 (0.56–1.10)	0.160	0.71 (0.48–1.04)	0.076
	AA	62 (20.5)	108 (23.0)	0.74 (0.49–1.12)	0.155	0.56 (0.35–0.90)	**0.017**
	CA+AA	209 (69.2)	350 (74.5)	0.77 (0.56–1.06)	0.111	0.66 (0.46–0.95)	**0.026**
**rs10069690**	CC	160 (54.6)	281 (61.4)	Reference		Reference	
	CT	107 (36.5)	157 (34.3)	1.20 (0.88–1.64)	0.261	1.41 (0.99–2.01)	0.058
	TT	26 (8.9)	20 (4.4)	2.28 (1.24–4.22)	**0.008**	2.22 (1.10–4.48)	**0.026**
	CT+TT	133 (45.4)	177 (38.6)	1.32 (0.98–1.78)	0.067	1.52 (1.08–2.12)	**0.017**

A = adenine; adj = adjustment for age; C = cytosine; CI = confidence interval; OR = odds ratio; Others = control group and cases with pleural plaques; SNP = single nucleotide polymorphism; T = thymine. Statistically significant values are printed in bold.

When the group of MM patients was compared with the cases with PP, polymorphic rs2736098 T allele remained statistically significantly associated with an increased risk of developing MM (CT genotype: OR = 1.63; CI = 1.18–2.24; p = 0.003; CT+TT genotype: OR = 1.46; CI = 1.08–1.99; p = 0.014) (Table 6). After adjustment for age, only CT genotype remained associated with significantly higher MM risk (OR_adj_ = 1.52; CI_adj_ = 1.06–2.16; p_adj_ = 0.022). Carriers of two polymorphic rs2736100 A alleles had a lower risk for developing MM, but only after adjustment for age (OR_adj_ = 0.58; CI_adj_ = 0.36–0.94; p_adj_ = 0.028). On the other hand, carriers of two polymorphic rs10069690 T alleles had a higher risk of MM development (OR = 2.10; CI = 1.12–3.96; p = 0.021). After adjustment for age, the risk for MM remained significant in carriers of two polymorphic alleles (OR _adj_ = 2.11; CI_adj_ = 1.02–4.34; p_adj_ = 0.043). Additionally, in the multivariable analysis the association was also significant in the dominant model (CT+TT genotype: OR _adj_ = 1.48; CI_adj_ = 1.05–2.10; p_adj_ = 0.027) (Table 6).

**TABLE 6.** Association between selected polymorphisms and risk of developing malignant mesothelioma (MM): comparison of patients with MM and cases with pleural plaques (PP)

**SNP**	**Genotype**	**OR (95% CI)**	**P**	**OR (95% CI)_adj_**	**P_adj_**
**rs2736098**	CC	Reference		Reference	
	CT	1.63 (1.18–2.24)	**0.003**	1.52 (1.06–2.16)	**0.022**
	TT	0.82 (0.45–1.51)	0.521	0.76 (0.38–1.53)	0.445
	CT+TT	1.46 (1.08–1.99)	**0.014**	1.37 (0.98–1.93)	0.069
**rs2736100**	CC	Reference		Reference	
	CA	0.84 (0.59–1.19)	0.330	0.77 (0.52–1.15)	0.196
	AA	0.78 (0.51–1.20)	0.257	0.58 (0.36–0.94)	**0.028**
	CA+AA	0.82 (0.59–1.15)	0.245	0.71 (0.48–1.03)	0.070
**rs10069690**	CC	Reference		Reference	
	CT	1.19 (0.86–1.65)	0.296	1.39 (0.96–2.01)	0.078
	TT	2.10 (1.12–3.96)	**0.021**	2.11 (1.02–4.34)	**0.043**
	CT+TT	1.30 (0.96–1.78)	0.096	1.48 (1.05–2.10)	**0.027**

A = adenine; C = cytosine; CI = confidence interval; OR = odds ratio; SNP = single nucleotide polymorphism; T = thymine. Statistically significant values are printed in bold.

### Treatment response rate in patients with malignant mesothelioma

The data on chemotherapy treatment and response are presented in Table 2. The majority of patients with MM received chemotherapy based on gemcitabine with cisplatin (N = 161; 57.3%). Complete and partial responses were achieved only in 3.9% and 28.5% of patients, respectively, while in 50.0% of patients, the disease was stable. Disease progression occurred in 17.6% of patients. The majority of patients thus had a poor chemotherapy response rate (N = 173; 67.6%).

We observed no significant associations between telomere length or their dynamics with a chemotherapy response rate (p > 0.05) (Table 7).

**TABLE 7.** Association between telomere length and chemotherapy response rate in patients with malignant mesothelioma (MM)

**Telomere length**	**Poor response Median (25–75%)**	**Good response Median (25–75%)**	**P**
A	1.20 (1.01–1.37)	1.28 (1–1.39)	0.576
B	1.21 (1.05–1.37)	1.28 (1.13–1.35)	0.601
C	1.29 (1.07–1.37)	1.23 (1.02–1.34)	0.369
Comparison B *vs*. A	0.04 (−0.1 to 0.1)	−0.01 (−0.11 to 0.07)	0.317
Comparison C *vs.* A	0.01 (−0.09 to 0.22)	−0.04 (−0.16 to 0.15)	0.241
Comparison C *vs.* B	0.03 (−0.09 to 0.13)	−0.01 (−0.12 to 0.07)	0.353

A = telomere length before first chemotherapy cycle; B = telomere length at third chemotherapy cycle; C = telomere length after completed chemotherapy or at disease progression

When we analysed the associations between *hTERT* polymorphisms and a chemotherapy response rate, only rs10069690 influenced the chemotherapy response rate in MM patients. Carriers of at least one polymorphic rs10069690 allele had a significantly better response rate to chemotherapy (CT genotype: OR = 1.18; CI = 1.03–3.17; p = 0.039; CT+TT genotype: RO = 1.72; CI = 1.00–2.93; p = 0.048). Both associations became even stronger after adjustment for weight loss and ECOG performance status (CT genotype: OR _adj_ = 2.08; CI_adj_ = 1.13–3.84; p _adj_ = 0.019; CT+TT genotype: RO_adj_ = 2.04; CI _adj_ = 1.13–3.67; p_adj_ = 0.017) (Table 8).

**TABLE 8.** Association between selected polymorphism and chemotherapy response rate in patients with malignant mesothelioma (MM)

**SNP**	**Genotype**	**Poor response N (%)**	**Good response N (%)**	**OR (95% CI)**	**P**	**OR (95% CI)_adj_**	**P_adj_**
**rs2736098**	CC	81 (68.6)	37 (31.4)	Reference		Reference	
	CT	76 (65.5)	40 (34.5)	1.15 (0.67–1.99)	0.611	1.20 (0.67–2.16)	0.542
	TT	11 (64.7)	6 (35.3)	1.19 (0.41–3.47)	0.745	1.27 (9.42–3.87)	0.671
	CT+TT	87 (65.4)	46 (34.6)	1.16 (0.68–1.96)	0.587	1.21 (0.69–2.14)	0.511
**rs2736100**	CC	53 (67.1)	26 (32.9)	Reference		Reference	
	CA	89 (70.6)	37 (29.4)	0.85 (0.46–1.55)	0.592	0.85 (0.45–1.63)	0.625
	AA	31 (60.8)	20 (39.2)	1.32 (0.63–2.74)	0.463	1.16 (0.53–2.57)	0.709
	CA+AA	120 (67.8)	57 (32.2)	0.97 (0.55–1.70)	0.911	0.94 (0.51–1.71)	0.831
**rs10069690**	CC	94(72.9)	35 (27.1)	Reference		Reference	
	CT	58 (59.8)	39 (40.2)	1.81 (1.03–3.17)	**0.039**	2.08 (1.13–3.84)	**0.019**
	TT	14 (66.7)	7 (33.3)	1.34 (0.50–3.60)	0.558	1.85 (0.64–5.31)	0.255
	CT+TT	72 (61.0)	46 (39.0)	1.72 (1.00–2.93)	**0.048**	2.04 (1.13–3.67)	**0.017**

A = adenine; Adj = adjustment for weight loss and ECOG performance status; C = cytosine; CI = confidence interval; OR = odds ratio; SNP = single nucleotide polymorphism; T = thymine. Statistically significant values are printed in bold.

### Survival of patients with malignant mesothelioma

Within the median follow-up time of the patients of 41.7 (22.8–77.3) months, median PFS was 10.0 (6.3–16.4) months and the median overall survival (OS) was 19.3 (10.0–30.3) months.

Telomere length or their dynamics were not associated with PFS, even after adjustment for CRP (all p > 0.05) (Table 9).

**TABLE 9.** Association between telomere length and progression-free survival in patients with malignant mesothelioma (MM)

**Telomere length**	**HR (95% CI)**	**P**	**HR (95% CI)_adj_**	**P_adj_**
A	2.15 (0.69–6.68)	0.185	1.66 (0.50–5.47)	0.408
B	1.02 (0.28–3.76)	0.976	0.92 (0.22–3.76)	0.905
C	1.69 (0.46–6.16)	0.430	1.49 (0.40–5.48)	0.551
Comparison B *vs.* A	0.16 (0.03–1.01)	0.052	0.23 (0.03–1.67)	0.145
Comparison C *vs.* A	1.01 (0.25–4.18)	0.985	1.36 (0.31–5.92)	0.682
Comparison C *vs.* B	1.57 (0.26–9.58)	0.624	2.16 (0.31–14.83)	0.435

Ad = adjustment for C-reactive protein (CRP); CI = confidence interval; HR = hazard ratio

**FIGURE 3.**
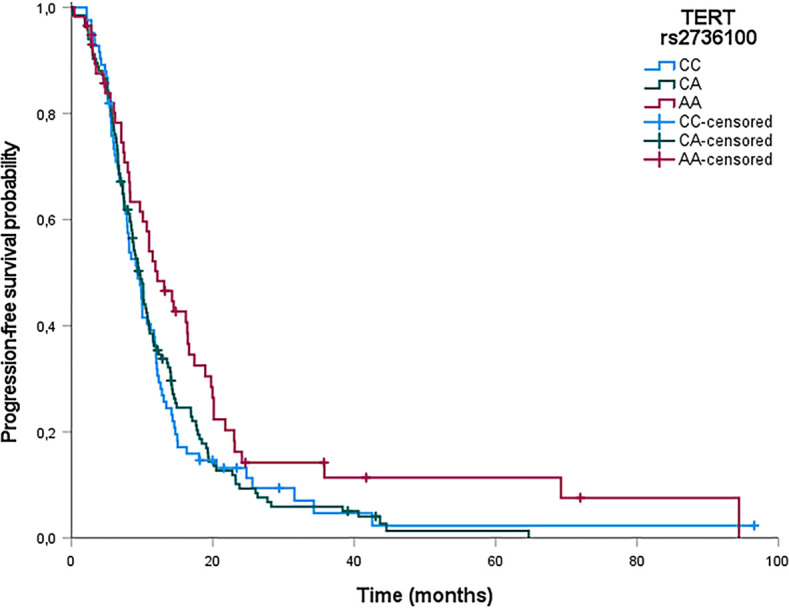
Kaplan Meier survival plot. Step = disease progression; cross = censored patient; AA = adenine-adenine; CA = cytosineadenine; CC = cytosine-cytosine; hTERT = telomerase reverse transcriptase

In Cox regression analysis (Table 10), patients with the rs2736100 AA genotype had significantly longer PFS compared to patients with the reference CC genotype (hazard ratio [HR] = 0.68; CI = 0.47–0.98; p = 0.038). The association of rs2736100 with PFS in patients with MM is illustrated as a function of time (Kaplan Meier plot) in Figure 3. None of other investigated polymorphisms were associated with PFS, not even after the adjustment for smoking status, asbestos exposure, weight loss, CRP level and histology type (p > 0.05) (Table 10).

**TABLE 10.** Association between selected polymorphisms and progression–free survival in patients with malignant mesothelioma (MM)

**SNP**	**Genotype**	**PFS Median (25%–75%)**	**HR (95% CI)**	**P**	**HR (95% CI)_adj_**	**P_adj_**
**rs2736098**	CC	10.2 (6.5–18.9)	Reference		Reference	
	CT	9.4 (6.1–14.5)	1.21 (0.93–1.57)	0.163	1.31 (0.98–1.76)	0.073
	TT	10.0 (6.6–14.2)	1.32 (0.79–2.20)	0.294	1.46 (0.82–2.59)	0.194
	CT+TT	9.7 (6.3–14.3)	1.22 (0.95–1.57)	0.127	1.33 (1.00–1.77)	0.051
**rs2736100**	CC	9.4 (5.9–13.4)	Reference		Reference	
	CA	9.7 (6.4–14.9)	0.98 (0.74–1.30)	0.879	0.83 (0.60–1.14)	0.250
	AA	12.2 (7.1–20.1)	0.68 (0.47–0.98)	**0.038**	0.68 (0.45–1.03)	0.070
	CA+AA	10.2 (6.5–17.6)	0.87 (0.67–1.15)	0.328	0.78 (0.57–1.06)	0.113
**rs10069690**	CC	10.7 (6.3–16.5)	Reference		Reference	
	CT	9.3 (6.1–15.0)	1.09 (0.83–1.42)	0.552	1.06 (0.79–1.43)	0.699
	TT	11.8 (7.3–13.4)	0.85 (0.52–1.38)	0.502	0.81 (0.48–1.38)	0.443
	CT+TT	9.4 (6.6–15.0)	1.04 (0.80–1.34)	0.791	1.01 (0.76–1.33)	0.963

A = adenine; Adj = adjustment for smoking, asbestos exposure, weight loss, C-reactive protein (CRP), and histology type of MM; C = cytosine; CI = confidence interval; HR = hazard ratio; PFS = progression free survival: SNP = single nucleotide polymorphism; T = thymine. Statistically significant values are printed in bold.

Additionally, the investigated polymorphisms were not associated with OS of patients with MM neither in univariable analysis, nor after the adjustment for asbestos exposure, ECOG performance status, CRP level, and histologic type (all p > 0.05) (Table 11).

**TABLE 11.** Association between selected polymorphisms and overall survival in patients with malignant mesothelioma (MM)

**SNP**	**Genotype**	**OS Median (25%–75%)**	**HR (95% CI)**	**P**	**HR (95% CI)_adj_**	**P_adj_**
**rs2736098**	CC	18.2 (10.1–28.6)	Reference		Reference	
	CT	19.3 (9.6–31.4)	1.01 (0.76–1.35)	0.944	1.02 (0.75–1.39)	0.899
	TT	24.4 (22.0–31.1)	0.84 (0.46–1.54)	0.573	0.94 (0.48–1.83)	0.859
	CT+TT	20.3 (9.9–31.2)	0.99 (0.75–1.31)	0.924	1.01 (0.75–1.36)	0.945
**rs2736100**	CC	17.5 (11.6–28.1)	Reference		Reference	
	CA	19.3 (10.7–32.5)	0.87 (0.64–1.20)	0.404	0.88 (0.62–1.24)	0.460
	AA	20.6 (9.6–31.4)	0.79 (0.53–1.17)	0.229	0.86 (0.56–1.31)	0.478
	CA+AA	19.5 (10.0–32.5)	0.85 (0.63–1.14)	0.270	0.87 (0.63–1.21)	0.410
**rs10069690**	CC	20.3 (9.6–31.4)	Reference		Reference	
	CT	19.3 (11.1–25.9)	1.09 (0.81–1.47)	0.578	1.13 (0.82–1.55)	0.455
	TT	16.0 (13.1–29.0)	1.01 (0.60–1.72)	0.958	0.74 (0.41–1.35)	0.321
	CT+TT	18.5 (11.4–29.0)	1.08 (0.81–1.43)	0.619	1.04 (0.77–1.41)	0.780

A = adenine; Adj = adjustment for asbestos exposure, ECOG performance status, C-reactive protein (CRP), histology type of MM; C = cytosine; CI = confidence interval; HR = hazard ratio; OS = overall survival; SNP = single nucleotide polymorphism; T = thymine

## Discussion

In our study, we evaluated whether telomere length or their dynamics and *hTERT* polymorphisms could serve as a biomarker for the risk of developing asbestos-related diseases, chemotherapy response, and progression in MM patients. Consistent with previous studies, we observed that patients with MM had shorter telomeres compared to cases with PP. Previous studies stated that the telomeres in cancer patients are shorter but stable when compared to healthy individuals.^18,20,21^ Also, a study analysing telomere length in pleural effusion cells reported shorter telomeres in 12 MM patients compared to 35 cases with non-neoplastic disease.^23^

Interestingly, older patients with MM had longer telomeres than younger patients with MM. According to our knowledge, telomere shortening is one of the most important markers of ageing.^19,33,34^ Furthermore, cancer cells have typically short telomeres^18,20,21^, as also shown for MM.^23^ However, the presence of telomerase reactivation in MM^22^ allows for an unlimited cell division potential and telomere length maintenance, which, on the contrary, does not occur in non-neoplastic cells,^35^ potentially contributing to the observed results.

In the first part of the study, we evaluated the association of *TERT* SNPs with a risk for MM. We observed rather consistent associations of the polymorphic rs2736098 T allele with an increased risk of MM in the additive or in the dominant genetic model. Although no specific studies on this association with MM have been conducted, recent studies have shown associations between rs2736098 and lung cancers^36,37^ as well as an increased risk for bladder cancer, while the risk was decreased for breast and colon cancers.^36^

Another important finding of this study was the decreased risk of MM associated with homozygosity for polymorphic rs2736100 A allele after adjustment for age. This observation emphasizes the importance of age as a contributing factor to carcinogenesis, although further investigation is needed to determine whether this association is coincidental. Our results are in agreement with previous studies indicating that carriers of two reference rs2736100 C alleles generally have a higher risk of developing idiopathic lung fibrosis, chronical obstructive pulmonary disease (COPD) and laryngeal cancer.^18,39,40^

Furthermore, we observed a significantly higher risk for MM in carriers of two polymorphic rs10069690 T alleles. To our knowledge, there are no other studies investigating this association in MM; however, rs10069690 has been linked to a higher overall cancer risk, specifically in breast, ovarian, lung and thyroid cancers.^41^

In the second part of the study, we evaluated the association of telomere length and *TERT* SNPs with a treatment outcome in MM. We did not find any associations between telomere length and MM chemotherapy response. While studies on breast cancer reported that chemotherapy can lead to telomere shortening in the short term, telomere length was shown to return to its pre-treatment level after two years.^42^ Given that our findings are based on a limited number of participants and that studies in MM patients are lacking, further analyses and investigations are necessary to gain a deeper understanding of the relationship between telomere length and a chemotherapy response in MM.

In our study, polymorphic rs10069690 T allele was associated with a good chemotherapy response. Moreover, this association became even more statistically significant after adjustment for age. Interestingly, our findings differ from a previous study in breast cancer, which is also telomerase-dependent cancer, where rs10069690 was associated with poor chemotherapy outcome.^43^ As there are currently no studies specifically exploring this relationship in MM, further studies are required to fully understand the impact of rs10069690 on a chemotherapy response in MM.

No significant associations were identified between telomere length and PFS in patients with MM. To our knowledge, there are no other studies that investigated the association between telomere length and survival in MM, and the existing survival analyses conducted for other cancer types yield contradictory results. An extensive study examining the effect of telomere length on survival in various benign and malignant diseases found no influence on cancer patients’ survival.^44^ Conversely, an American study on pancreatic cancer observed that shorter telomeres were linked to poorer OS, while *hTERT* polymorphisms had no statistically significant impact on OS.^45^ Similarly, our study showed no significant associations between the investigated *hTERT* polymorphisms and overall survival of MM patients. Due to the inconsistent knowledge in this field, further studies should be performed to better define the factors influencing the outcome of MM.

Finally, our study has shown that carriers of two polymorphic rs2736100 A alleles had a lower risk for MM progression. However, this area of research is still limited, and our finding contrasts with a kidney cancer study that identified rs2736100 as an independent factor associated with a poor prognosis.^46^ Similarly, an Indian study reported that rs2736100 contributes to a poorer prognosis of glioma patients.^47^ On the other hand, a Chinese study did not seem to validate the relationship between this polymorphism and a poor prognosis in papillary thyroid carcinoma.^48^ It is essential to consider that the mentioned studies did not focus on MM and were not conducted on Caucasians, thus caution should be applied when interpreting the data. Therefore, future studies investigating these associations with a specific focus on MM are required.

In conclusion, our results suggest that telomere length and genetic polymorphisms in the *hTERT* gene have a limited role as a biomarker for the risk of developing asbestos-related diseases. Collectively, our study did not demonstrate the role of telomere length as a biomarker for a MM chemotherapy response; however, with a cautious interpretation, *hTERT* polymorphisms may represent a biomarker for the chemotherapy outcome in MM. Similarly, telomere length does not seem to impact PFS, while *hTERT* polymorphisms may be used as a biomarker for the risk of MM progression. So far, our findings have been encouraging, yet further studies are necessary to validate these associations in independent patient cohorts and elucidate the role of telomere length and genetic variants of the *hTERT* gene in MM.
